# Curcumin and Glu-GNPs Induce Radiosensitivity against Breast Cancer Stem-Like Cells

**DOI:** 10.1155/2020/3189217

**Published:** 2020-12-28

**Authors:** Ke Yang, Zhiwei Liao, Yujian Wu, Mengjie Li, Tingting Guo, Jiayi Lin, Yanwu Li, Chenxia Hu

**Affiliations:** ^1^School of Pharmaceutical Science, Guangzhou University of Chinese Medicine, Guangzhou 510006, China; ^2^Guangzhou Medical University Affiliated Cancer Hospital, Guangzhou 510635, China; ^3^Science and Technology Innovation Center, Guangzhou University of Chinese Medicine, Guangzhou 510006, China

## Abstract

Breast cancer stem cells are an important cause of radiotherapy resistance in the clinical treatment of breast cancer patients. How to target breast cancer stem cells is the key to improving the efficacy of breast cancer radiotherapy. We proposed for the first time that curcumin combined with glucose nanogold particles (Glu-GNPs) targeted breast cancer stem cells to reduce radiotherapy resistance, which can significantly enhance the apoptosis level of MCF-7 and MDA-MB-231 breast cancer stem-like cells (BCSCs) after radiotherapy and antiproliferation and colony-forming. Under simulated hypoxic conditions, curcumin combined with Glu-GNPs can significantly improve the ROS level of MCF-7 and MDA-MB-231 mammospheres; reduce the expression of HIF-1*α* and HSP90, thereby inhibiting the tumor cells' own stress ability; promote the apoptosis of tumor stem cells; and enhance the sensitivity of radiotherapy. The current results indicate that the combination of curcumin and Glu-GNPs has great potential to relieve tumor hypoxia and increase radiosensitivity on BCSCs, providing scientific research data for developing a novel radiosensitizer with high efficiency and low toxicity.

## 1. Introduction

Breast carcinoma is the most prevalent female malignancy and has high mortality rate, only next to lung cancer. Rebecca et al. [1] predicted the number of new cases of infiltrating carcinoma in America in 2019. There will be nearly 268,600 new cases of breast malignancy accounting for 30% of all new cancer diagnoses, and 41,760 deaths are from breast cancer accounting for 15% of all cancer deaths in women.

Although the currently available treatments may eliminate the primary tumor, many patients still die from tumor relapse and metastasis. Accumulating evidence indicates that a minor population of special cells in cancer tissue, which are similar to normal stem cells, acts as an important part to tumor formation and growth. Their characteristics of self-renewal, strong proliferation capacity, multidifferentiation potential, and strong oncogenicity indicate that they may be the source of tumorigenesis, relapse, and metastasis of cancer. Radiation therapy is one of the conventional methods to treat breast cancer and has showed great efficacy in clinical practice. However, breast cancer recurrence and metastasis after radiotherapy and the development of radioresistance after repeated irradiations represent a major challenge.

In 2003, Al-Hajj et al. [2] firstly isolated stem cell-specific surface marker CD44^+^CD24^-/low^ cells from breast tumor tissue. Many studies have indicated that BCSCs exhibit stronger resistance to radiotherapy and chemotherapy compared with ordinary breast cancer cells [3–6]. Phillips et al. [7] used the clone formation test to detect cell survival fractions at 2 Gy irradiation (SF2); it was observed that SF2 was higher in MCF-7 and MDA-MB-231 stem cells (0.46 and 0.69, respectively) compared to their respective adherent cells (0.2 and 0.5, respectively). The levels of *γ*H2AX and ROS suggested that the microsphere stem cells were less prone to DNA damage. It was the first time that BCSCs were confirmed to have strong radiotherapy resistance characteristics. In view of the radiotherapy resistance leading to cancer relapse and metastasis, new treatment methods targeting cancer stem cells are crucial for inhibiting the development of breast cancer.

Gold nanoparticles (GNPs) are a type of nanomaterial approved for clinical trials by FDA in the US. It was demonstrated that, when nanometal particles were irradiated by X-ray, the effect of ionizing radiation on the tumor cells was enhanced, which promoted the release of free radicals, damaging DNA and inducing cell apoptosis [8–11]. Our previous study showed that Glu-GNPs were assimilated in MCF-7 adherent cells and THP-1 suspension cells more easily than GNPs [12] It suggested that glucose tagging may be a right way for promoting the uptake of GNPs in tumor cells, as tumor cells can take up more glucose than normal cells. In addition, it was previously demonstrated that these nanometal particles enhanced killing effects on tumor cells without increasing damage to the surrounding normal tissues in a mouse model, thereby reducing the adverse effects of radiotherapy [13, 14].

Curcumin is an active compound extracted from the underground rhizome of the tumeric plant. It has widespread function in preventing and treating tumor. There are some accumulating evidences indicating that curcumin can enhance the radiosensitivity in several types of cancer [15–17]. Our studies have demonstrated that curcumin inhibited mammosphere formation, characteristics of stem cells in BCSCs [18]. We herein aimed to investigate whether curcumin and Glu-GNPs can enhance the radiosensitivity in BCSCs and the underlying mechanism.

In our study, we confirmed the inhibiting effects on cell proliferation and clone formation of curcumin and Glu-GNPs, alone and in combination, in both types of spheres after X-ray irradiation. The radiosensitivity was further enhanced by treatment with curcumin combined with Glu-GNPs. Under hypoxic conditions, the curcumin group, the Glu-GNP group, and the combination group all showed G0/G1 cell cycle arrest. Induction of apoptosis and increased ROS levels were observed in spheres either irradiated with X-ray alone or treated with a combined application of curcumin, Glu-GNPs, and X-ray. Furthermore, curcumin and Glu-GNPs downregulated the HIF-1*α* and HSP90 expression in both types of tumor spheres.

## 2. Materials and Methods

### 2.1. Compound and Chemicals

Curcumin (purity ≥ 77%), thiopolyethylene glycol (thio-PEG), and 1-thio-d-glucose (Glu) were bought from Sigma-Aldrich, Germany. CoCl_2_ was purchased from AMRESCO (Cleveland, OH, USA).

### 2.2. Adherent Cell and Tumor Sphere Culture

MCF-7 and MDA-MB-231 cells were obtained from ATCC (Manassas, VA, USA). They were cultivated in Dulbecco's modified Eagle's medium (DMEM) containing 10% FBS and 1% P/S (Gibco, USA) in an incubator with 5% CO_2_ at 37°C. Then, according to the culture method reported by Dontu et al. [19, 20], the two kinds of breast cancer cells were plated in ultralow attachment 6-well plates with SFM including DMEM-F12 (Gibco), P/S, bFGF, EGF, B27 (Cyagen, USA), insulin, and BSA (Sigma-Aldrich). The growth medium was replenished every 2 days with fresh medium. Cells cultured under the condition forming nonattached tumor spheres were digested once a week. Third-generation spheres were collected to detect the CD44^+^CD24^-/low^ cells by FCM (BD Accuri™ C5, USA).

### 2.3. Synthesis of Glu-GNPs and Cell Uptake

Referring to our previously described method [12], the complex of thio-PEG and glucose was added to the solution of GNPs (Beijing DK Nanotechnology Co., Ltd., Beijing, China) and well-mixed to obtain the final solution of Glu-GNPs. The sample was performed by centrifugation for 30 min (9000 g, 4°C) to remove supernatant. The cell uptake of GNPs and Glu-GNPs was examined using transmission electron microscopy (TEM). MDA-MB-231 spheres (1 × 10^6^/per well) were seeded in ultralow attachment 6-well plates for 24 h. The cells were starved for 6 h by changing the medium without serum. The drug groups were treated with 0.1% GNPs or Glu-GNPs (20 *μ*L per 1 mL medium) for 6 h, and the controls were treated with deionized water. The spheres were fixed by 2.5% glutaraldehyde, dehydrated, and paraffin embedded, and ultrathin sections were cut and examined by TEM.

### 2.4. Cell Treatment

Single-cell suspensions obtained from MCF-7 and MDA-MB-231 spheres were inoculated at 4 × 10^5^ per well in 6-well plates. After treatment with 30 *μ*M curcumin for 24 h, cells were starved in the medium without serum for 6 h. DMSO was used as a control. The cells were then treated with 0.1% Glu-GNPs (20 *μ*L per 1 mL medium). After 6 h, the cells were washed with PBS and then supplemented with fresh medium containing serum.

### 2.5. Irradiation

Irradiation was performed with a 6 MV X-ray at a total dose of 0 and 4 Gy using the two-photon medical linear accelerator Elekta Synergy 3630 (Elekta Instrument AB, Stockholm, Sweden). The dose rate was 2 Gy/min, with radiation for two minutes. The depth of the treatment was 2 cm, and the irradiation distance is 50 cm.

### 2.6. Cell Viability and Clone Formation Test

After they were irradiated by X-ray, cell suspensions of the two types of spheres were separately inoculated (2.5 × 10^4^ per well) in 96-well plates and incubated. Afterwards, 10 *μ*L CCK-8 solution (EnoGeneCell Counting Kit-8; EnoGene Biotech Co., NY, USA) was added to each well and incubated for 3 h. The absorbance of each well was measured using a PerkinElmer EnSpire Reader at 490 nm (Perkin Elmer, Waltham, MA, USA). The cell viability of the treated cells was expressed relative to that of the cells treated with DMSO only. All values are presented as the mean ± standard deviation of at least triplicate samples. Meanwhile, the spheres were suspended in DMEM containing serum and inoculated in 6-well plates (500 cells per well). All the cells were cultured in the medium for 14 d. The colonies were fixed with paraformaldehyde, then stained with Giemsa for 30 min. The clones were observed and counted. The experiment was repeated 3 times, and the average was taken.

### 2.7. Hypoxic Treatment

Chemical hypoxia was induced with 100 *μ*mol/mL CoCl_2_ on the two types of spheres for 2 h prior to X-ray. Under hypoxic conditions, cell cycle distribution, apoptosis rates, and ROS levels in each group were detected by FCM using BD Accuri™ C5 (BD Biosciences).

### 2.8. Cell Apoptosis Detection

Apoptotic rate was assessed referring to the protocol of the double-staining cell apoptosis kit (BestBio, China). After 12 h of irradiation under hypoxic conditions, the spheres were digested and washed with PBS, centrifuged, and suspended in a 500 *μ*L binding buffer (1 × 10^6^ cells/mL). The spheres were stained with Annexin-V-FITC (50 *μ*g/mL, 5 *μ*L) followed by the addition of PI (50 *μ*g/mL, 10 *μ*L). The samples were analyzed by FCM.

### 2.9. Cell Cycle Detection

After 12 h of irradiation under hypoxic conditions, the mammospheres were collected and digested into a single-cell suspension with 0.05% trypsin and washed twice with PBS, then centrifuged for 5 min at 800 g, and suspended in 500 *μ*L cold PBS and fixed with 70% ethanol at 4°C for 12 h. The cells (1 × 10^6^ cells/mL) were treated with RNase solution (80 mg/mL, 20 *μ*L) and propidium iodide (50 *μ*g/mL, 400 *μ*L) for 1 h. The stained cells were assessed by FCM.

### 2.10. ROS Level Analysis

ROS levels in the irradiated cells were detected with a ROS assay kit (Thermo Fisher Scientific). After irradiation, the spheres were cultured with a new medium for 12 h, digested, and centrifuged. Cells were washed with PBS and suspended in a 400 *μ*L ROS assay buffer. Cell suspension (1 × 10^6^ cells/mL) was treated with 1x ROS Assay Stain while avoiding light to incubate for 1 h at 37°C and then analyzed by FCM.

### 2.11. mRNA Expression Analysis

Total RNA was extracted by using a TRIGene kit (GenStar Biosolutions, China), and cDNA was synthesized from 1 mg of total RNA according to the protocol. The primer sequences are shown in Table 1. The quantitative PCR kit (Takara Biomedical Technology Co., China) was used for real-time PCR analysis.

### 2.12. Protein Expression Analysis

Total proteins from the MCF-7 and the MDA-MB-231 sphere were extracted and measured, and the BCA protein quantitative kit (Beyotime, China) was used to quantify the protein concentration. Rabbit anti-human HIF-1*α* (Abcam, ab243860) and rabbit anti-human HSP90 (Cell Signaling Technology, #4877) monoclonal antibodies (1 : 1,000) were used for incubation overnight at 4°C. Secondary antibodies (1 : 2,000) for 1 h at room temperature. Western blot images were detected with a Tanon 5200 Multi Chemiluminescent Imaging system (Tanon Science and Technology, China), and ImageJ software (National Institutes of Health, Bethesda, MD, USA) was used to analyze the gray values of each group.

### 2.13. Statistical Analysis

Statistical analyses were performed using an Anova test (one way or two way depending upon the type of data) in GraphPad software. The results were presented as the mean ± standard deviation of triplicate experiments, and *P* < 0.05 was considered to indicate a statistically significant difference.

## 3. Results

### 3.1. CD44^+^CD24^−^ Cells Were Increased in Tumor Mammospheres

**Figure 1:**
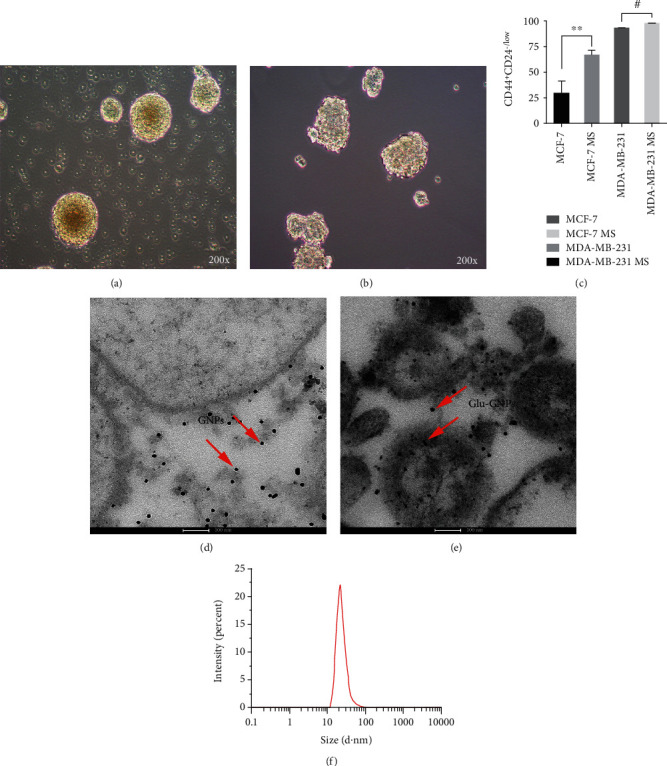
Cell morphology and cell intake of GNPs, Glu-GNPs in MDA-MB-231 MS by TEM: (a) MCF-7 MS; (b) MDA-MB-231 MS; (c) CD44^+^CD24^-/low^ cells were increased in two types of spheres; (d) GNPs; (e) Glu-GNPs; (f) size for the Glu-GNPs measured by NanoSight. ^∗∗^*P* < 0.01 and ^#^*P* < 0.05. TEM: transmission electron microscopy; GNPs: gold nanoparticles; Glu-GNPs: GNPs with glucose; MS: mammospheres.

MCF-7 and MDA-MB-231 adherent cells were cultured with SFM, and the third-generation spheres were harvested (Figure 1(a) and 1(b)). As CD44^+^CD24^-/low^ is a key marker of BCSCs, the CD44^+^CD24^−^ subpopulation in the two types of adherent cells and spheres was detected by FCM. The proportion of CD44^+^CD24^−^ cells was significantly increased in MCF-7 spheres (66.75 ± 0.05%) compared with MCF-7 adherent cells (29.7 ± 0.12%), *P* < 0.01; in addition, the biomarker of MDA-MB-231 spheres increased from 94.50 ± 0.02% to 98.35 ± 0.01% (*P* < 0.05) (Figure 1(c)).

### 3.2. Cellular Uptake of Glu-GNPs in Mammospheres

After TEM observation, it was found that both GNPs and Glu-GNPs were absorbed by MDA-MB-231 spheres, especially Glu-GNPs which had more uptake than GNPs, as seen in Figure 1(d) and 1(e). The uptake distribution of MCF-7 cells for GNPs and Glu-GNPs has been published [12].

### 3.3. Effects of Curcumin Combined with Glu-GNPs on Cell Viability in Mammospheres

**Figure 2:**
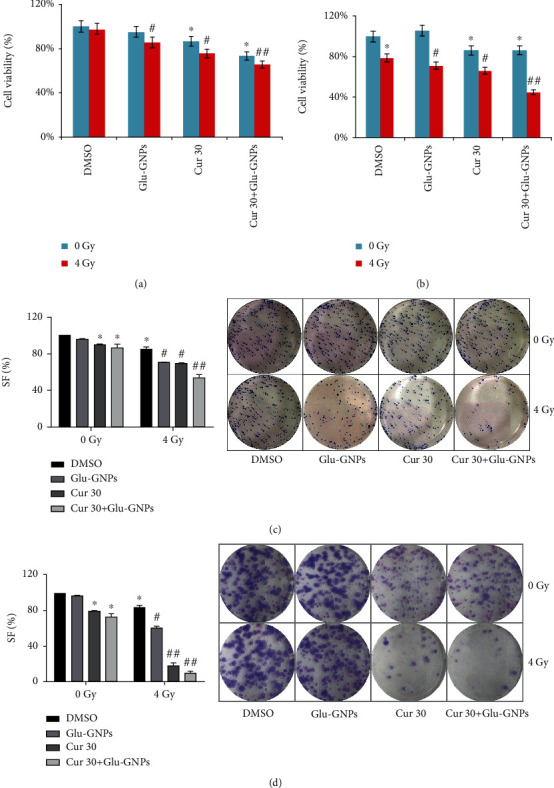
Cell viability and clone-forming capability was suppressed by curcumin and Glu-GNPs with 0 or 4 Gy radiotherapy. Viability was measured in (a) MCF-7 MS and (b) MDA-MB-231 MS. Survival fraction (SF) and colonies of (c) MCF-7 MS and (d) MDA-MB-231 MS. The data were normalized to the control. ^∗^*P* < 0.05 vs. control at 0 Gy; ^#^*P* < 0.05 and ^##^*P* < 0.01 vs. control at 4 Gy.

Curcumin and Glu-GNPs, alone and in combination, exerted inhibition on the two types of spheres after 4 Gy irradiation (*P* < 0.05). The radiosensitivity was further enhanced by treatment with curcumin combined with Glu-GNPs (*P* < 0.01). With 0 Gy irradiation, curcumin alone and curcumin combined with Glu-GNPs decreased viability on both kinds of spheres (*P* < 0.05), and there is no statistical difference between curcumin alone and curcumin combined with Glu-GNPs, *P* > 0.05 (Figure 2(a) and 2(b)). Curcumin itself was effective in inhibiting viability of spheres, and its combination with Glu-GNPs enhanced the effectiveness of radiotherapy.

### 3.4. Clone Formation Ability Was Repressed by Curcumin Combined with Glu-GNPs

Without irradiation, the curcumin group and the curcumin in combination with Glu-GNP group exerted antiproliferation on the two types of spheres. After irradiation with 4 Gy, curcumin and Glu-GNPs, alone and in combination, inhibited the clone-forming ability in the two kinds of spheres (*P* < 0.05). Curcumin showed inhibition of colony formation in MDA-MB-231 spheres significantly after 4 Gy irradiation (*P* < 0.01). The radiosensitivity was further enhanced by treatment with curcumin combined with Glu-GNPs in both types of spheres (*P* < 0.01; Figure 2(c) and 2(d)).

### 3.5. Effects of Curcumin and Glu-GNPs on Spheres under Hypoxic Condition

**Figure 3:**
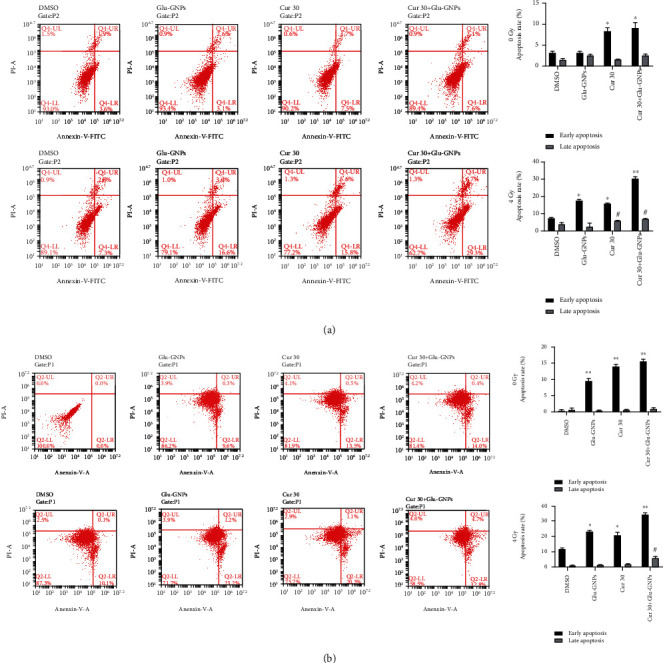
Curcumin and Glu-GNPs with radiotherapy promoted apoptosis rate of breast cancer spheres. The apoptotic rates were detected by FCM in (a) MCF-7 MS and (b) MDA-MB-231 MS. ^∗^*P* < 0.05 vs. DMSO group at early apoptosis; ^#^*P* < 0.05 and ^##^*P* < 0.01 vs. DMSO group at late apoptosis.

**Figure 4:**
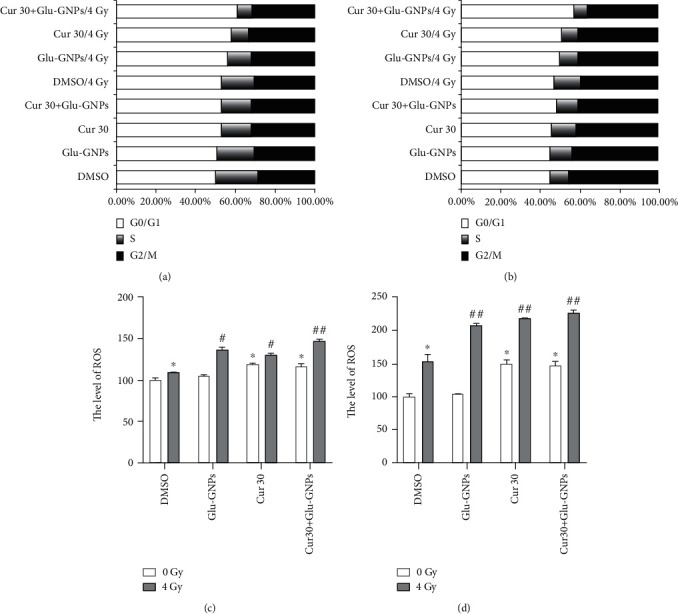
Cell cycle distribution and ROS levels were altered by curcumin and Glu-GNPs with radiotherapy in two types of spheres by FCM. The cell cycle distribution was detected in (a) MCF-7 MS and (b) MDA-MB-231 MS; detection of ROS levels in (c) MCF-7 MS and (d) MDA-MB-231 MS. The data were normalized to the control. ^∗^*P* < 0.05 indicates significance compared to the control (DMSO-treated group) with 0 Gy radiation treatment; ^#^*P* < 0.05 indicates significance compared to the control (DMSO-treated group) with 4 Gy radiation treatment. ROS: reactive oxygen species.

The apoptosis of cells was further enhanced by treatment with curcumin combined with Glu-GNPs (*P* < 0.01). The apoptosis level was increased by curcumin and curcumin with Glu-GNPs after 0 Gy in both MCF-7 and MDA-MB-231 mammospheres (*P* < 0.05; Figure 3). MCF-7 and MDA-MB-231 mammospheres were irradiated after CoCl_2_ treatment (100 *μ*mol/mL). The results demonstrated that the apoptosis level and G0/G1 phase cells were increased in the curcumin group and the curcumin in combination with Glu-GNP group in the two types of spheres without irradiation (Figure 4). After 4 Gy irradiation, curcumin and Glu-GNPs, alone and in combination, promoted cell apoptosis and G0/G1 phase arrest in both kinds of spheres. The apoptosis of cells and the number of G0/G1 phase cells were further enhanced by treatment with curcumin combined with Glu-GNPs (Figure 4(a) and 4(b)). Meanwhile, ROS levels in each group increased after irradiation especially in the curcumin combined with Glu-GNP group (*P* < 0.01; Figure 4(c) and 4(d)).

### 3.6. HIF-1*α* and HSP90 mRNA Expression Are Affected by Curcumin and Glu-GNPs

**Figure 5:**
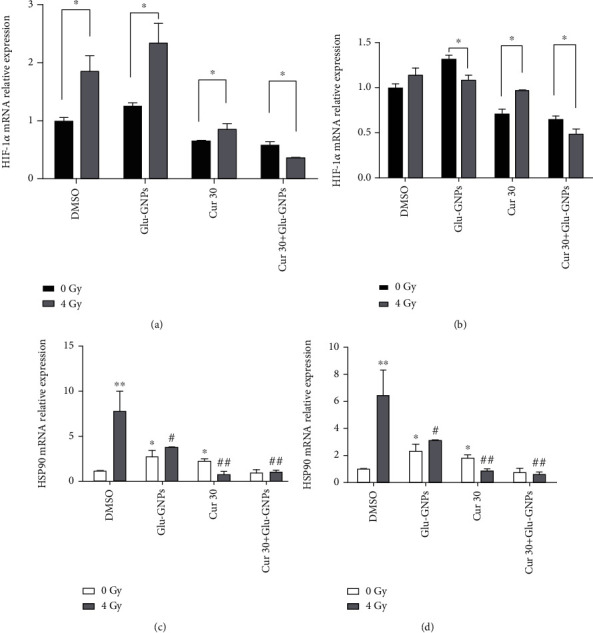
HIF-1*α* and HSP90 mRNA expression in breast cancer spheres was regulated by curcumin and Glu-GNPs with radiotherapy. mRNA level of HIF-1*α* in (a) MCF-7 MS and (b) MDA-MB-231 MS; mRNA level of HSP90 genes in (c) MCF-7 MS and (d) MDA-MB-231 MS. *β*-Actin was used as the reference gene. ^∗^*P* < 0.05 indicates significance compared to the control (DMSO-treated group) with 0 Gy radiation treatment; ^#^*P* < 0.05 indicates significance compared to the control (DMSO-treated group) with 4 Gy radiation treatment.

We were surprised to find a notable increment of the HSP90 mRNA level in the two kinds of spheres of the DMSO group after 4 Gy irradiation (*P* < 0.01). It was estimated that HSP90 is a kind of stress protein which will produce greatly when the cancer cells are stimulated by hypoxia and radiation or other physical and chemical factors to protect themselves. Curcumin alone or curcumin combined with Glu-GNPs effectively reduced the increase of HIF-1*α* and HSP90 mRNA level caused by hypoxia or radiation in the two types of spheres (*P* < 0.05, Figure 5).

### 3.7. Protein Level of HIF-1*α* and HSP90 Are Affected by Curcumin and Glu-GNPs

**Figure 6:**
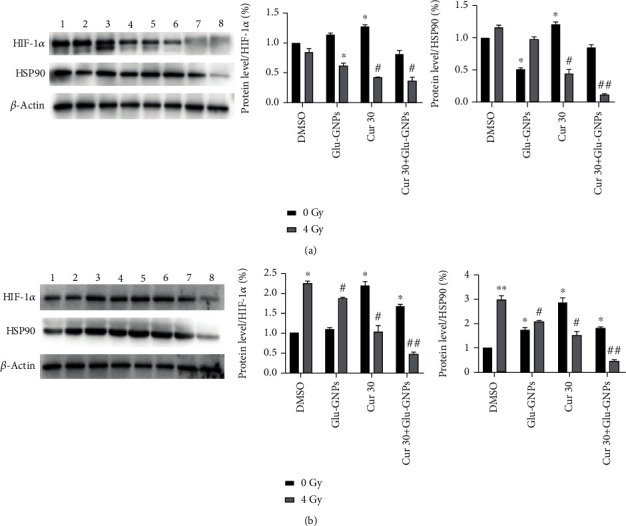
HIF-1*α* and HSP90 protein levels were regulated by curcumin and Glu-GNPs with radiotherapy in two types of spheres. (a) MCF-7 MS and (b) MDA-MB-231 MS treated with 0 or 4 Gy; 1, 2, 3, and 4 (5, 6, 7, and 8) represent, respectively, DMSO, Glu-GNPs, Cur 30, and Cur 30 combined with Glu-GNPs irradiated with 0 (or 4) Gy. The data were expressed as a ratio of density of individual proteins to the expression of *β*-actin.

Without irradiation, curcumin and curcumin combined with Glu-GNPs increased HIF-1*α* and HSP90 protein levels in both types of spheres (*P* < 0.05). However, after 4 Gy irradiation, both curcumin and Glu-GNPs decreased the HIF-1*α* and HSP90 protein production in both kinds of spheres, respectively (*P* < 0.05). The HIF-1*α* and HSP90 protein expression in the cells decreased further by treatment with curcumin combined with Glu-GNPs (*P* < 0.01; Figure 6).

## 4. Discussion

Curcumin is widely used as a food seasoning in some Asian countries. A great number of studies have investigated its chemopreventive activities or anticancer function on several types of cancer [21–24]. In our previous studies, we found that curcumin reduced the invasion and migration of MDA-MB-231 adherent cells as well as tumor sphere formation, stemness, and EMT process in BCSCs. In recent years, it was investigated that curcumin has a potential to enhance the effect of radiotherapy in breast cancer [25–27]. However, whether curcumin has radiosensitizing effects on BCSCs and the underlying mechanism remain unclear. In addition, our studies have demonstrated that GNPs may be a promising radiosensitizer which exerts a radiosensitizing effect on MCF-7 adherent cells and THP-1 suspension cells. Therefore, it is critical to continue to determine if curcumin could enhance the radiosensitivity of GNPs in BCSCs and to uncover the mechanisms of its radiosensitizing effect.

We selected two kinds of breast cancer cells of different differentiating degree, MCF-7 and MDA-MB-231. Tumor spheres were cultured by the suspension method, and the CD44^+^CD24^-/low^ subpopulation, which is recognized as a special stem-like phenotype of breast cancer, was significantly increased in spheres, which was consistent with our previous findings [18]. In the study, we found that curcumin and Glu-GNPs, alone and in combination, exerted antiproliferation and clone-forming inhibition on both types of spheres after X-ray irradiation. Curcumin combined with Glu-GNPs significantly improved the sensitivity to radiotherapy in spheres, suggesting that curcumin and Glu-GNPs exert a synergistic sensitizing effect.

As an unstable molecule, ROS can cause DNA damage and activate oncogenes to promote cancer which is generally considered as one of the main reasons in the development of malignant tumors, and antioxidant agents can repress the cancerogenesis and metastasis through scavenging ROS. However, on the other hand, some studies showed that the hypoxic region in solid tumors with the decrease of the ROS level lacked oxygen free radicals to damage DNA, leading to radiation resistance of cancer cells and reducing the radiotherapy efficacy.

We continued to investigate if curcumin or Glu-GNPs can affect the ROS level to induce apoptosis and cell cycle changes in BCSCs under hypoxic conditions. Firstly, we used CoCl_2_ to treat spheres to make a hypoxic cell model. Then, under hypoxic conditions, cell cycle distribution, apoptosis rates, and ROS concentration in each group were detected by FCM. We found that 4 Gy irradiation alone enhanced the quantity of G0/G1 phase cells and apoptosis rates in two types of spheres. Meanwhile, ROS levels increased significantly after curcumin and Glu-GNP treatment. Under 4 Gy radiation, exposure of tumor spheres to the combination group of curcumin and Glu-GNPs led to significant increase in the ROS level, apoptosis induction, and G0/G1 phase cells. Furthermore, recent research showed both endogenous and exogenous ROS can mediate DNA damage, multiple apoptotic pathways, and cell cycle arrest to inhibit cancer cells [28–30]. Therefore, we propose that the different effects of ROS on tumor development may be related to the tumor staging and radiation, which is worthy of further study.

Based on that, we further explore the underlying mechanism of their radiosensitizing effect in two types of spheres. HIF-1*α*, as an oxygen-sensitive transcriptional activator, plays a crucial role in tumor survival, progression, and metastasis in hypoxic conditions [31]. The hypoxic microenvironment also provides the basis to cancer stem cells which are involved in cancer metastasis and resistance to therapy [32, 33]. The increase of the HIF-1*α* expression can also lead to the reduction of the radiotherapy effect. D. Pagoulatos et al. [34, 35] demonstrated that the activity of HSP90 can affect the expression level of HIF-1*α*, thereby regulating the apoptosis of tumor cells. The expression level of the HSP90 protein may be used as an index of radiosensitivity. In the study, the spheres with hypoxic treatment irradiated by 4 Gy X-ray alone led an increase in HIF-1*α* and HSP90 mRNA and protein levels. Curcumin and Glu-GNPs lowered the level of both HIF-1*α* and HSP90. Furthermore, curcumin acts synergistically with Glu-GNPs to exert more prominent effects. The results suggest that the combination of curcumin and Glu-GNPs have great potential to relieve tumor hypoxia and increase the radiosensitivity on breast cancer stem-like cells.

## 5. Conclusions

Curcumin combined with Glu-GNPs can significantly enhance the radiosensitivity of human breast cancer MCF-7 and MDA-MB-231 mammospheres. Its molecular mechanism may be related to inhibiting the expression of HIF-1a and HSP90, increasing the ROS level and inducing apoptosis of cancer cells.

## Figures and Tables

**Table 1:** Reverse transcription-quantitative polymerase chain reaction.

Genes	Primers (5′-3′)
*β*-Actin	F: CTCCATCCTGGCCTCGCTGT
	R: GCTGTCACCTTCACCGTTCC
HIF-1*α*	F: ACGTTCCTTCGATCAGTTGTCACC
	R: GGCAGTGGTAGTGGTGGCATTAG
HSP90	F: CCAGTTCGGTGTTGGTTTTTAT
	R: CAGTTTGGTCTTCTTTCAGGTG

## Data Availability

The data used to support the findings of this study are included within this article.

## Abbreviations

ATCC: American Type Culture Collection
BCSCs: Breast cancer stem-like cells
bFGF: Basic fibroblast growth factor
CCK-8: Cell counting kit-8
DMEM: Dulbecco's modified Eagle's medium
DMSO: Dimethyl sulfoxide
EGF: Epidermal growth factor
EMT: Epithelial-mesenchymal transition
FBS: Fetal bovine serum
FCM: Flow cytometry
FDA: Food and Drug Administration
Glu: 1-Thio-d-glucose
Glu-GNPs: Gold nanoparticles with glucose
GNPs: Gold nanoparticles
MS: Mammospheres
PBS: Phosphate-buffered saline
PEG: Polyethylene glycol
ROS: Reactive oxygen species
SF: Survival fraction
SFM: Serum-free medium
TBS: Tris-HCl-buffered saline
TEM: Transmission electron microscopy.
