# Correction to “Curcumin and Glu‐GNPs Induce Radiosensitivity against Breast Cancer Stem‐Like Cells”

**DOI:** 10.1155/bmri/9816203

**Published:** 2025-11-19

K. Yang, Z. Liao, Y. Wu, M. Li, T. Guo, J. Lin, Y. Li, C. Hu, “Curcumin and Glu‐GNPs Induce Radiosensitivity against Breast Cancer Stem‐Like Cells,” *BioMed Research International*, https://doi.org/10.1155/2020/3189217

In the article, an error was introduced in Figure 3a during the production process, whereby the bottom row DMSO and Glu‐GNPs plots are duplicates. The correct Figure 3 is shown as follows:

Figure 3Curcumin and Glu‐GNPs with radiotherapy promoted apoptosis rate of breast cancer spheres. The apoptotic rates were detected by FCM in (a) MCF‐7 MS and (b) MDA‐MB‐231 MS.  ^∗^ *p* < 0.05 vs. DMSO group at early apoptosis; ^#^ *p* < 0.05 and ^##^ *p* < 0.01 vs. DMSO group at late apoptosis.

**Figure:**
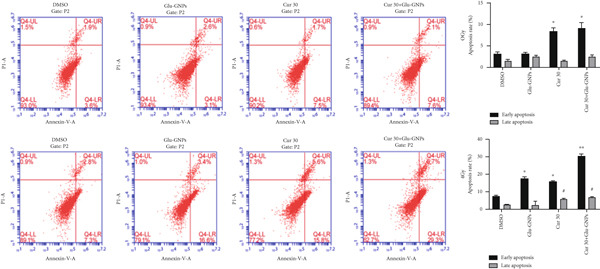
(a)

**Figure:**
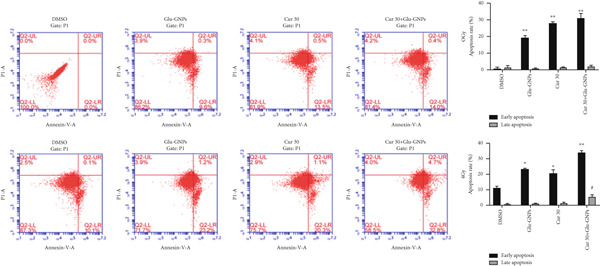
(b)

We apologize for this error.

